# Multi-Objective Optimization of Resistance Welding Process of GF/PP Composites

**DOI:** 10.3390/polym13152560

**Published:** 2021-07-31

**Authors:** Guowei Zhang, Ting Lin, Ling Luo, Boming Zhang, Yuao Qu, Bangke Meng

**Affiliations:** 1 School of Materials Science and Engineering, Beihang University, Beijing 100191, China; zgwhitbuaa@yeah.net (G.Z.); luoling_buaa@yeah.net (L.L.); zbm@buaa.edu.cn (B.Z.); 2 Design and Development Center, AECC Commercial Aircraft Engine Co., Ltd., Shanghai 201104, China; 3 School of Chinese Medicine, Heilongjiang Academy of Chinese Medical Sciences, Harbin 150036, China; quyuao@126.com; 4 Technology Department, JOY Composites Co., Ltd., Tai’an 271033, China; mbk@bjcomposites.com

**Keywords:** resistance welding, thermoplastic composites (TPCs), glass-fiber-reinforced polypropylene (GF/PP), multi-objective optimization

## Abstract

Thermoplastic composites (TPCs) are promising materials for aerospace, transportation, shipbuilding, and civil use owing to their lightweight, rapid prototyping, reprocessing, and environmental recycling advantages. The connection assemblies of TPCs components are crucial to their application; compared with traditional mechanical joints and adhesive connections, fusion connections are more promising, particularly resistance welding. This study aims to investigate the effects of process control parameters, including welding current, time, and pressure, for optimization of resistance welding based on glass fiber-reinforced polypropylene (GF/PP) TPCs and a stainless-steel mesh heating element. A self-designed resistance-welding equipment suitable for the resistance welding process of GF/PP TPCs was manufactured. GF/PP laminates are fabricated using a hot press, and their mechanical properties were evaluated. The resistance distribution of the heating elements was assessed to conform with a normal distribution. Tensile shear experiments were designed and conducted using the Taguchi method to evaluate and predict process factor effects on the lap shear strength (LSS) of GF/PP based on signal-to-noise ratio (S/N) and analysis of variance. The results show that current is the main factor affecting resistance welding quality. The optimal process parameters are a current of 12.5 A, pressure of 2.5 MPa, and time of 540 s. The experimental LSS under the optimized parameters is 12.186 MPa, which has a 6.76% error compared with the result predicted based on the S/N.

## 1. Introduction

Continuous fiber-reinforced composite materials are widely used in aerospace, architecture, ships, and automobiles because of their high specific strength, high specific stiffness, lightweightness, and designability. Fiber-reinforced composites can be classified into thermoset composites (TSCs) and thermoplastic composites (TPCs) owing to the difference in the resin matrix. Although the mechanical properties of TPCs differ from those of traditional TSCs, TPCs offer advantages of rapid prototyping [1], reprocessing, and environmental recycling [2]. Hence, TPCs are increasingly being valued and used in practical industries [3,4].

The connection assembly of TPC components is unavoidable in applications. Traditional mechanical connections can cause issues, such as stress concentration, galvanic corrosion, and material damage [5,6]. Adhesive connections require complicated surface treatments, and the curing cycle is relatively long [7,8,9]. The hot-melt connection can effectively prevent these problems. Based on the heating method, fusion welding can be categorized into hot plate, hot gas, ultrasonic and radio signals, microwave, resistance welding, laser, and induction. Among them, resistance welding is regarded as one of the most promising welding methods because of insufficient surface treatment, simplicity, low cost of equipment, reprocessing, and the potential for online control [10]. The principle of resistance welding is to apply Joule’s law to convert electrical energy into heat, and Joule’s Law equation is as follows:

(1) $$E=I^{2}Rt,$$

where *E* is the energy, *I* the electric current, *R* the resistance, and *t* the time of current passing. When the electric current passes through the heating element, the heating element heats up, and the generated energy exceeds the heat loss of the material and the surrounding environment. Consequently, the temperature of the joint begins to increase. When the temperature increases to a certain point, the thermoplastic resin matrix begins to melt and diffuse at the interface. Subsequently, the current is discontinued, and the joint begins to cool with sufficient pressure to achieve bonding. Therefore, the quality of the resistance welding is determined by the current, time, and pressure [11].

The resistance welding of TPCs has been reported in the literature. Hou et al. [12] investigated the lap shear strength (LSS) of carbon fiber-reinforced polyetherimide (CF/PEI) specimens welded at four different powers and reported that the power level significantly affected the LSS, and that an appropriate power level caused the LSS to reach its peak value, whereas excessive power decreased the LSS. Ageorges et al. [13] monitored the pressure and displacement fluctuations during displacement control and pressure control in a study regarding CF/PEI and reported six processing stages of resistance welding, i.e., the initial compaction, thermal expansion, melt flow, cooling, solidification, and contraction stages. Li and Zhang [14] reported that the cooling rate affected the LSS of carbon fiber-reinforced poly-ether-ether-ketone (CF/PEEK). The lower the cooling rate, the better is the LSS of the joint. Vincent and Louis [15] reported that different ambient temperatures affected the LSS of carbon fiber-reinforced polyphenylene sulfide (CF/PPS), and that the LSS decreased linearly from the ambient temperature of 20 °C–105 °C. Lorena and Samia [16] tested welded joints of glass fiber-reinforced polyphenylene sulfide (GF/PPS) and reported that fractographic analyses were effective for evaluating welding quality. Currently, studies regarding the application of resistance welding technology in TPCs primarily focus on the application of CF-or GF-reinforced high-performance resins such as PEEK, PEI, and PPS in aerospace. Glass fiber-reinforced polypropylene (GF/PP) TPCs are typical and low-cost thermoplastic composite materials, which have broad prospects for civil use. Research pertaining to CF/PP and GF/PP thermoplastic composites has been reported, albeit few.

The heating element is the source of heat generated at the welding interface and remains in the joint after welding. Therefore, it significantly affects the quality of the resistance welding process. The material type, quality, and size of the heating element significantly affect the quality and mechanical properties of welded joints. Carbon fiber prepregs and metal meshes are the two main types of heating elements. When carbon-fiber-reinforced thermoplastics are welded, carbon fiber prepregs can maintain their compatibility with plastic materials; however, the fibers are easily damaged during welding, thereby resulting in an uneven temperature distribution. Some studies have focused on the use of carbon fibers as heating elements [17,18,19,20]. Generally, metal mesh (typically stainless-steel mesh) improves the temperature uniformity of the welding area [21], thereby improving the welding performance and affording a wider processing window. Therefore, most researchers prefer to use stainless steel meshes [22,23,24,25].

The Taguchi method is a low-cost and high-efficiency quality control method that involves orthogonal experiments for robust parameter design to minimize fluctuations in product design parameters [26,27,28,29,30,31]. Analysis of variance (ANOVA) [32,33,34,35,36,37] is a typically used method of data analysis, in which the total dispersion of the experimental data is decomposed into a dispersion derived from different factors and data are estimated to discover the importance of each factor in the total dispersion. 

The aim of this study is to propose an optimization method for resistance welding based on GF/PP TPCs and stainless-steel mesh heating elements. First, a self-designed resistance welding equipment suitable for the resistance welding of TPCs was manufactured. Second, the Taguchi method was used for orthogonal experimental design and conducting tensile shear experiments to reveal the effects of process parameters (welding current, welding time, and welding pressure) on the LSS of GF/PP joints, and the signal-to-noise ratio (S/N) method was used to evaluate the LSS of GF/PP joints. Finally, ANOVA was used to identify the optimized process parameters, the S/N method was applied to predict the LSS under the optimized process parameters, and the prediction results were verified experimentally. This study provides a reference for the resistance welding of TPCs.

## 2. Experimental Procedure

### 2.1. Materials

The GF/PP TPC sheet used in this experiment was provided by China Guangdong Nuclear Power Juner New Material Co., Ltd. The preparation process involved arranging the prepregs based on the requirements of the layup and setting them on the mold. Compression molding was used to manufacture GF/PP laminates. The GF/PP prepregs were heated to 180 °C for 3 min, and a pressure of 3 MPa was maintained until the GF/PP plate was cooled and shaped. Subsequently, the GF/PP plate was cut into 100 mm × 25 mm rectangular strips. The GF/PP plate parameters are presented in Table 1.

### 2.2. Mechanical Properties

The mechanical properties of the GF/PP composites were tested based on ASTM D3039, as shown in Table 2.

### 2.3. Heating Element

**Figure 1:**
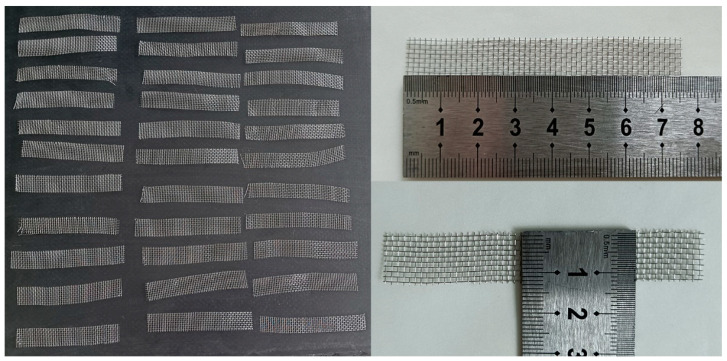
Stainless steel mesh heating elements.

**Figure 2:**
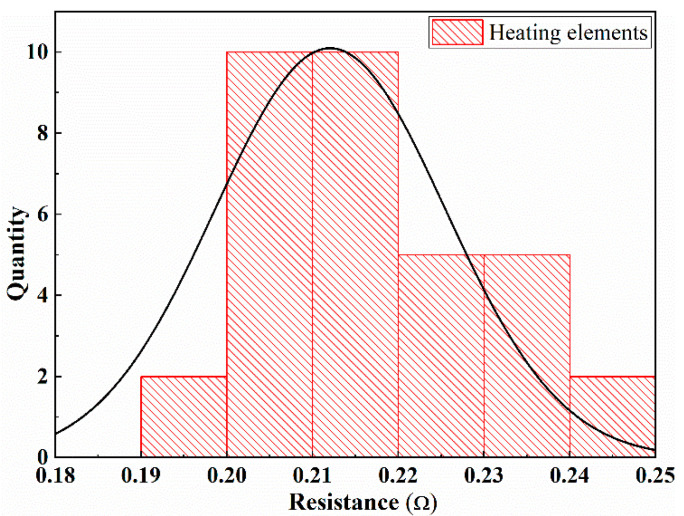
Resistance distribution of heating element.

The heating elements used in this study were made of a 20-mesh 304 stainless steel; as such, they can withstand high temperatures and are not easy to oxidize. The wire diameter and aperture size of the 304 stainless steel mesh were 0.23 and 1.5 mm, respectively. The heating element size was 12 ± 0.5 mm × 75 mm, as shown in Figure 1. The heating element generates heat through the thermal effect of the resistance. Therefore, the resistance of the heating elements must be measured. The resistance values of the heating elements are presented in Figure 2. As shown, the values ranged between 0.19 and 0.24 Ω, which conformed to the normal distribution. The resistance values of the heating elements selected in the study were between 0.20 and 0.23 Ω.

### 2.4. Experiments

**Figure 3:**
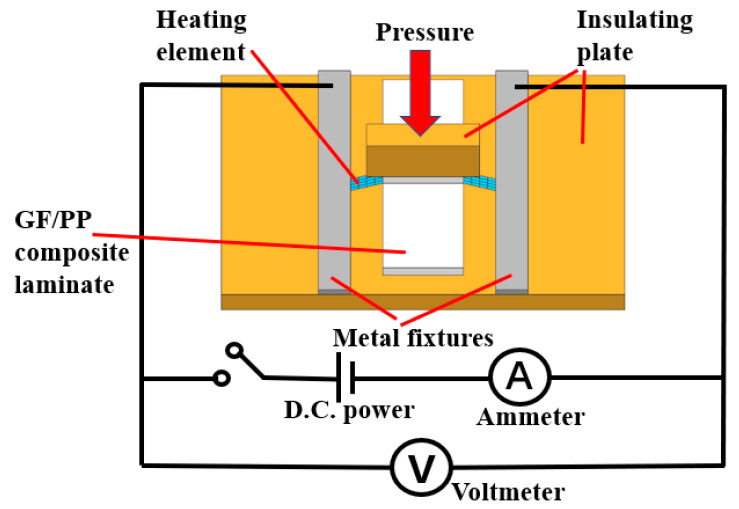
Schematic diagram of resistance welding device.

**Figure 4:**
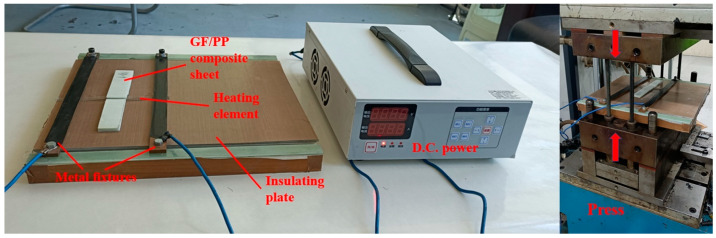
Self-designed resistance welding device.

**Figure 5:**
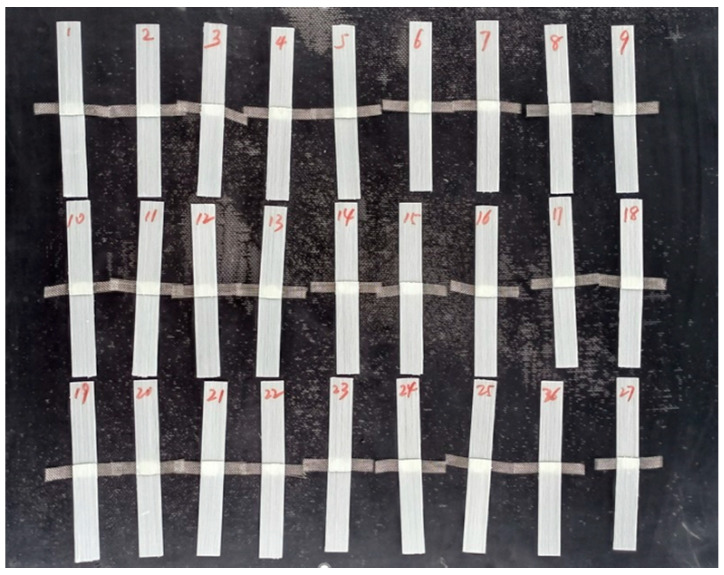
Photograph of test samples.

**Figure 6:**
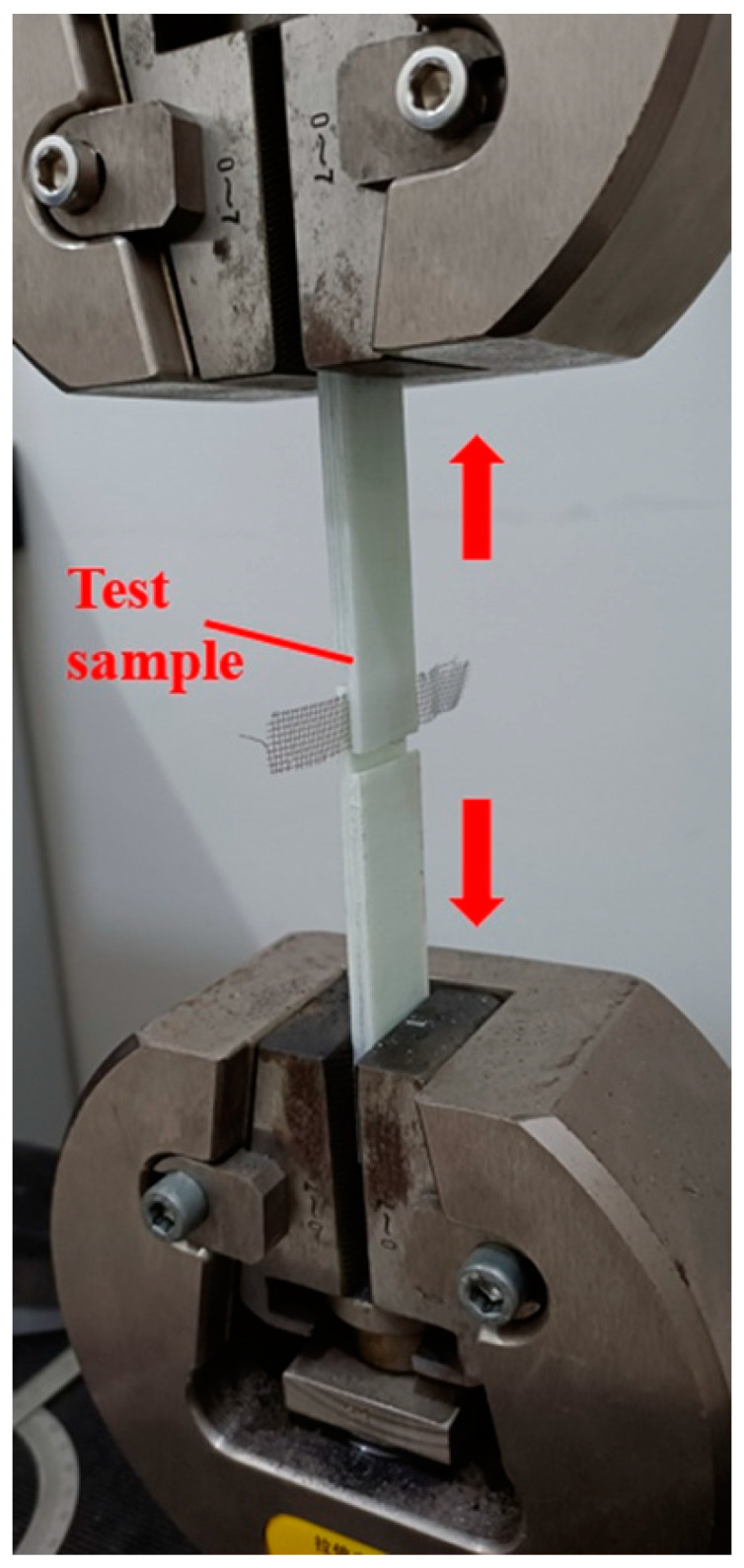
Test setup.

A schematic diagram of a typical experimental device for the resistance welding of TPCs is shown in Figure 3. Figure 4 shows the self-designed resistance welding device used in this experiment. The samples were processed using a welding device. The heating element was sandwiched in the middle of the GF/PP composite laminates. The two sides of the heating element were connected to the two metal fixtures, which were fixed on the insulating plate by screws. Subsequently, the circuit was switched on, and the current parameters were set on the power supply device. The required pressure was applied through the press, and a resistance welding process was performed. Figure 5 shows the prepared tensile shear samples. For the resistance welding of TPCs, the three factors of input current, welding pressure, and heating time were investigated to determine the effect of the LSS of GF/PP joints. The welding quality was evaluated using the LSS and S/N values of the GF/PP sample. The experiments were performed based on the ASTM D1002 standard. In the tensile shear test, the sample was installed on the fixture of the testing machine to ensure that the sample was on the centerline of the fixture. The loading rate of the test was 2 mm/min. The process of the sample tensile shear test is illustrated in Figure 6. 

## 3. Parameter Setting and Orthogonal Table Construction

### 3.1. Evaluation and Prediction Method

The S/N method is a practical evaluation and prediction method, and the S/N conversion equation is as follows [38]:

(2) $$S/N\;\mathrm{ratio}\left(\eta \right)=-10\log_{10}\left[\frac{1}{n}\overset{n}{\underset{i=1}{\sum }}\;\frac{1}{y_{i}^{2}}\right],$$

where *η* is the S/N (units: dB); *y_i_* is the experimental observation value, which is the tested LSS value in this study; *n* is the number of repetitions of each experiment. 

The S/N prediction equation is as follow:

(3) $$\eta_{p}=\eta_{m}+\overset{j}{\underset{i=1}{\sum }}\left(\eta_{f}-\eta_{m}\right),$$

where *η_p_* is the predicted S/N value, *η_m_* the mean S/N value of all experiments, *η_f_* the S/N value at the optimal level of a factor, and *j* the number of factors.

First, the LSS was converted into the S/N value using Equation (2), and the optimal process parameters were determined through an ANOVA of the S/N. Second, the S/N value under the optimal process parameters was predicted using Equation (3). Finally, the predicted S/N value was converted back to the predicted LSS using Equation (2). The predicted LSS was verified experimentally.

### 3.2. Orthogonal Experimental Design

Taguchi’s method uses mature orthogonal tables to conduct multifactor and multilevel experiments and investigate the degree of effect of various factors on the experimental results. It is advantageous because it can reduce the number of experiments to the maximum extent without affecting the results through scientific design, while conserving the test time and costs. Furthermore, it is beneficial to investigate the effects of various factors and levels on the indicators and select the best combination of process parameters. For the resistance welding process of the TPCs, three factors (input current, welding pressure, and heating time) were investigated for their effects on the LSS, and each factor was set at four levels. The welding quality was evaluated using the LSS and S/N values of the sample. Considering the selection factors of the orthogonal table and the experimental investigation factors, an orthogonal table comprising three factors and four levels was selected (L16). The design of the L16 standard orthogonal table is presented in Table 3. Among them, factor A was the input current, and the input current levels were set to 10.5, 11.5, 12.5, and 13.5 A; factor B was the welding pressure, and the pressure levels were set to 1.0, 1.5, 2.0, and 2.5 MPa; factor C was the welding time, and the time levels were set to 180, 360, 540, and 720 s, as shown in Table 4. Table 5 shows the specific level combination of each factor and the experimental values of the mean LSS and S/N.

### 3.3. ANOVA

In the ANOVA, the S/N of the sample was the dependent variable, whereas the current, pressure, and time were independent variables. As shown in Table 5, the greater the LSS and S/N, the better the quality of resistance welding of the GF/PP composites, indicating that the corresponding factors (current, pressure, and time) were the optimal process parameters. ANOVA is based on the total sum of the squared deviations of the observed variables. If the proportion of the sum of squared deviations between groups is large, it implies that the changes in the observed variables are primarily caused by the control variables, which can be explained based on the control variables. The control variable significantly affects the observation variable; by contrast, if the proportion of the sum of squared deviations between groups is small, then it implies that the change in the observation variable is not primarily caused by the control variable. The different levels of control variables do not significantly affect the observed variables, and the changes in the values of the observed variables are caused by random variable factors.

## 4. Results and Discussion

### 4.1. Study Regarding PROCESS Parameters

During the resistance welding of GF/PP, the LSS of the joint is generally less than the strength of the base material (GF/PP). This implies that the joint is the weakest link in the entire structure; hence, a higher welding strength is pursued during the welding. Table 6 shows the ANOVA of the S/N; as shown, the current, pressure, and time exhibit statistical and physical significance. Among them, the current was the main factor, and its contribution rate was 58.12%. The contribution rates of the welding time, pressure, and experimental error were 23.07%, 15.29%, and 3.51%, respectively.

**Figure 7:**
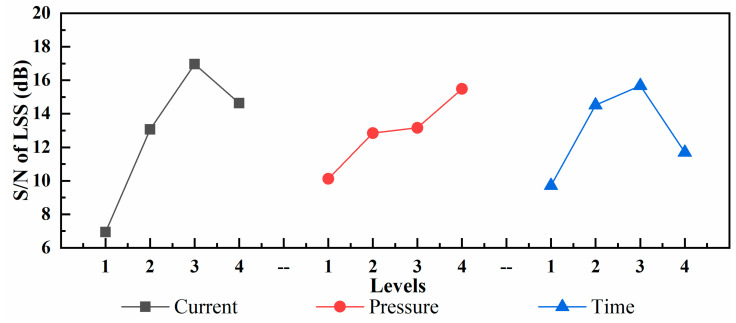
Mean S/N value under different levels of various factors.

Figure 7 shows the changes in the mean S/N value under different levels of various factors. The input current and welding time simultaneously determine the heat provided by the power supply. When the current level is low or the welding time is short, the heat generated by the resistance cannot provide the required energy for the plastic to soften and flow completely; therefore, the welding strength is low. However, when the current is extremely high or the welding time is extremely long, the energy and temperature in the joint will further increase, thereby causing the plastic to become yellow and deteriorate. Simultaneously, the plastic flow drives the movement of the fibers, resulting in a decrease in the joint quality. The role of pressure in the welding process is to promote the movement of molten plastics. When the pressure increases, the PP can penetrate each other more effectively, thereby improving the welding strength. In addition, it was observed experimentally that the combination of the optimal levels of process parameters was A3B4C3; the optimal LSS was obtained when the current was 12.5 A, pressure was 2.5 MPa, and welding time was 540 s. It can be predicted that the LSS of GF/PP under the optimal combination was 13.07 MPa based on Equations (2) and (3). The combination of the optimal parameters was determined, although not shown in Table 5. Hence, experiments should be conducted to verify the optimal parameters.

**Figure 8:**
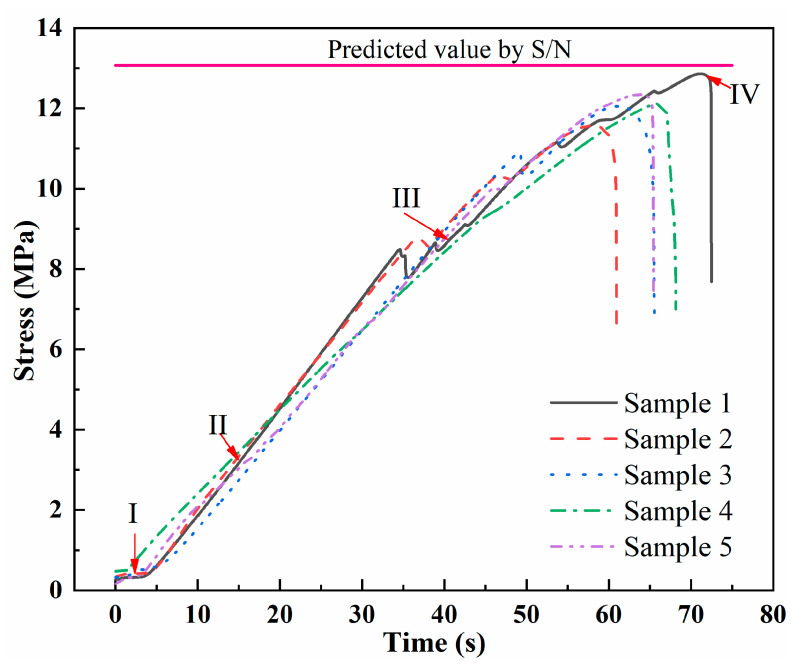
Time–stress relationship of GF/PP sample.

Figure 8 shows the typical time–stress curve of the resistance welding sample of GF/PP. Taking sample 1 curve as an example, the failure of the lap joint of GF/PP can be classified into four stages: (I) Pre-stretching stage: the mechanical testing machine through fine-tuning results in an accurate clamping between the tensile fixture and the specimen in the axial alignment state and, at this time, as the displacement increases, the increase in force is extremely slow, and the change in stress with strain is not apparent; (II) the stress growth stage: as the stretching process progresses, the stress increases rapidly with strain, and then cracks appear; (III) the modulus decline stage: with the appearance and expansion of cracks, the modulus of the bonding interface decreases, but the overall sample is not damaged; (IV) the failure stage: with the growth and accumulation of microcracks, failure occurs at the bond. In the experiments, the mean LSS of the samples was 12.186 MPa, which had an error of 6.76% compared with the predicted LSS value.

### 4.2. Destruction MODE Analysis

**Figure 9:**
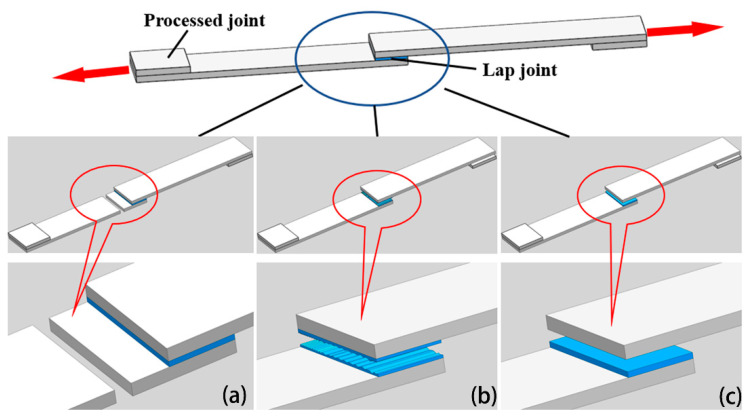
Typical failure modes of lap joints: (**a**) Coupon failure; (**b**) Intralaminar failure; (**c**) Interface failure.

**Figure 10:**
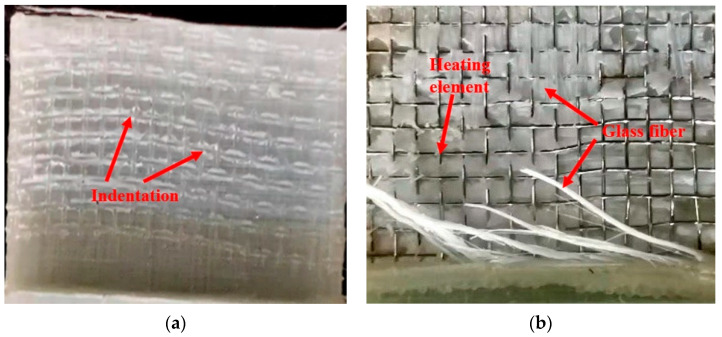
Macro appearance of lap joints: (**a**) Current 10.5 A, pressure 1 MPa, and time 180 s; (**b**) Current 12.5 A, pressure 2.5 MPa, and time 540 s.

Three typical failure modes of lap joints (see Figure 9) exist: coupon failure, intralaminar failure, and interface failure. In coupon failure, the welding strength is much higher than the strength of the base material, i.e., the base material is destroyed before the lap joint is destroyed, which is the ideal case. Typically, only intralaminar and interface failures are observed. If the lap joint shows intralaminar failure, then welding is considered successful. Figure 10a shows the interface failure of the lap joint at the current level of 10.5 A, pressure of 1 MPa, and time of 180 s. Only the indentation of the stainless-steel metal mesh was observed in the bonding area, which shows that the PP only softens and does not penetrate each other under the low-level current, time, and pressure; hence, the welding quality of the lap joint is inferior. Figure 10b shows the intralaminar failure of the lap joint at the current level of 12.5 A, pressure of 2.5 MPa, and time of 540 s. The failure is primarily caused by the fracture and pull-out of the GF in the bonding area, and it was observed that the heating element embedded well into the interface. In the two bonding surfaces of the sample, the PP was fully melted and penetrated mutually; therefore, good bonding was achieved and the welding strength improved.

## 5. Conclusions

In this study, an optimization method for the resistance welding process was proposed based on GF/PP TPCs, and the effects of the process control parameters were investigated. The following conclusions were obtained:

1. A self-designed resistance welding platform containing a DC power supply, an electrode, a heating element, an adherend, an insulation board, and a pressure control unit was established. The resistance values of the heating elements for the resistance welding process conformed to the normal distribution.
2. Using the Taguchi method and ANOVA, it was discovered that among the three process factors (welding current, welding pressure, and welding time) of the GF/PP resistance welding process, current was the main factor affecting the resistance welding quality of GF/PP TPCs, with a contribution rate of 58.12%. The contribution rates of time, pressure, and experimental error were 23.07%, 15.29%, and 3.51%, respectively.
3. The S/N method was used to establish a relationship between the LSS and process factors to optimize the LSS. Among the different level combinations of various factors, the optimal process parameters were a current of 12.5 A, pressure of 2.5 MPa, and time of 540 s. The error between the experimental and predicted LSS values was 6.76%.
4. The primary failure mode was intralaminar failure, which was primarily caused by fiber fracture and pull-out, and the damaged surface of the sample indicated that the heating element can be well implanted in the laminate, revealing good bonding at the interface.

## Figures and Tables

**Table 1:** GF/PP laminate parameters.

Parameter	Value
Thickness	2.4 mm
Layup	[0/90/90/0]_2_
Fiber weight content	60%

**Table 2:** Mechanical properties of GF/PP composites.

Properties	Value
0° Tensile strength	479.26 MPa
0° Tensile modulus	29.64 GPa
90° Tensile strength	13.71 MPa
90° Tensile modulus	3.53 GPa

**Table 3:** Standard orthogonal table.

No.	Factor A (Current)	Factor B (Pressure)	Factor C (Time)
1	1	1	1
2	1	2	2
3	1	3	3
4	1	4	4
5	2	1	2
6	2	2	1
7	2	3	4
8	2	4	3
9	3	1	3
10	3	2	4
11	3	3	1
12	3	4	2
13	4	1	4
14	4	2	3
15	4	3	2
16	4	4	1

**Table 4:** Factors and levels.

Factors	Levels			
	1	2	3	4
Current/A	10.5	11.5	12.5	13.5
Pressure/MPa	1	1.5	2.0	2.5
Time/s	180	360	540	720

**Table 5:** Input variables and experimental results.

No.	Parameters			Performance Characteristics	
	Factor A (Current/A)	Factor B (Pressure/MPa)	Factor C (Time/s)	Mean LSS (MPa)	S/N Value (dB)
1	1(10.5)	1(1)	1(180)	1.01	0.09
2	1(10.5)	2(1.5)	2(360)	2.4	7.60
3	1(10.5)	3(2)	3(540)	3.42	10.68
4	1(10.5)	4(2.5)	4(720)	2.93	9.34
5	2(11.5)	1(1)	2(360)	4.21	12.49
6	2(11.5)	2(1.5)	1(180)	3.54	10.98
7	2(11.5)	3(2)	4(720)	3.16	9.99
8	2(11.5)	4(2.5)	3(540)	8.73	18.82
9	3(12.5)	1(1)	3(540)	6.85	16.71
10	3(12.5)	2(1.5)	4(720)	6.57	16.35
11	3(12.5)	3(2)	1(180)	5.25	14.40
12	3(12.5)	4(2.5)	2(360)	10.47	20.40
13	4(13.5)	1(1)	4(720)	3.6	11.13
14	4(13.5)	2(1.5)	3(540)	6.65	16.46
15	4(13.5)	3(2)	2(360)	7.57	17.58
16	4(13.5)	4(2.5)	1(180)	4.65	13.35
				*η_m_*	12.90

**Table 6:** ANOVA of the S/N values.

Variables	Level-1	Level-2	Level-3	Level-4	SS	df	Mean Variance	F-Test	P
Current	6.93	13.07	16.97	14.63	220.87	3	73.622	33.109	58.12
Pressure	10.11	12.85	13.16	15.48	58.11	3	19.37	8.711	15.29
Time	9.71	14.52	15.67	11.70	87.67	3	29.223	13.142	23.07
Errors					13.34	6	2.224		3.51
Total					379.99	15			100

SS, sum of squares; df, degrees of freedom; P, contribution.

## Data Availability

Not applicable.
